# Primary Aortoenteric Fistula in a Patient With Prior Abdominopelvic Surgery and Opioid-Induced Constipation

**DOI:** 10.7759/cureus.34895

**Published:** 2023-02-12

**Authors:** Akash Patel, Adewale Ajumobi, Jerry Chang

**Affiliations:** 1 Internal Medicine, Eisenhower Health, Rancho Mirage, USA; 2 Gastroenterology and Hepatology, Eisenhower Health, Rancho Mirage, USA; 3 Internal Medicine, University of California Riverside School of Medicine, Riverside, USA; 4 Interventional Radiology, Eisenhower Health, Rancho Mirage, USA

**Keywords:** hematochezia, endovascular stenting, aorto-enteric fistula, abdomino-pelvic surgery, endovascular procedure, massive gastrointestinal bleeding, opiods induced constipation

## Abstract

Aortoenteric fistulas (AEF) represent a rare, life-threatening cause of gastrointestinal bleeding with an incidence of 0.007 per million. Iliac artery-enteric fistulas represent an even more uncommon variant of AEFs. Prompt diagnosis and intervention are required to prevent associated morbidity and mortality. Herein, we report a rare case of iliac-enteric fistula in a patient with hematochezia.

## Introduction

Aortoenteric fistulas (AEF) is an abnormal connection between aortic vessels and the gastrointestinal lumen. AEF is a rare but catastrophic cause of gastrointestinal bleeding. The rarity of the disorder, the absence of specific symptoms, and confounders such as NSAID usage, and incidental ulcers on upper endoscopy can cause a delay in diagnosis [1,2].

The aetiology of AEF is assumed to be due to continual irritation between the intestinal wall and the aortic branches, which leads to the gradual development of aortic luminal ulceration and, ultimately, fistula formation [3]. In many cases, a history of aortic repair surgery leads clinicians to suspect AEF. However, AEF can develop in the context of prior abdominopelvic surgery and enteric physical or inflammatory stress [3,4]. Here, we describe a case of haematochezia due to life-threatening AEF in a patient with a history of bladder surgery and opioid-induced constipation.

## Case presentation

**Figure 1:**
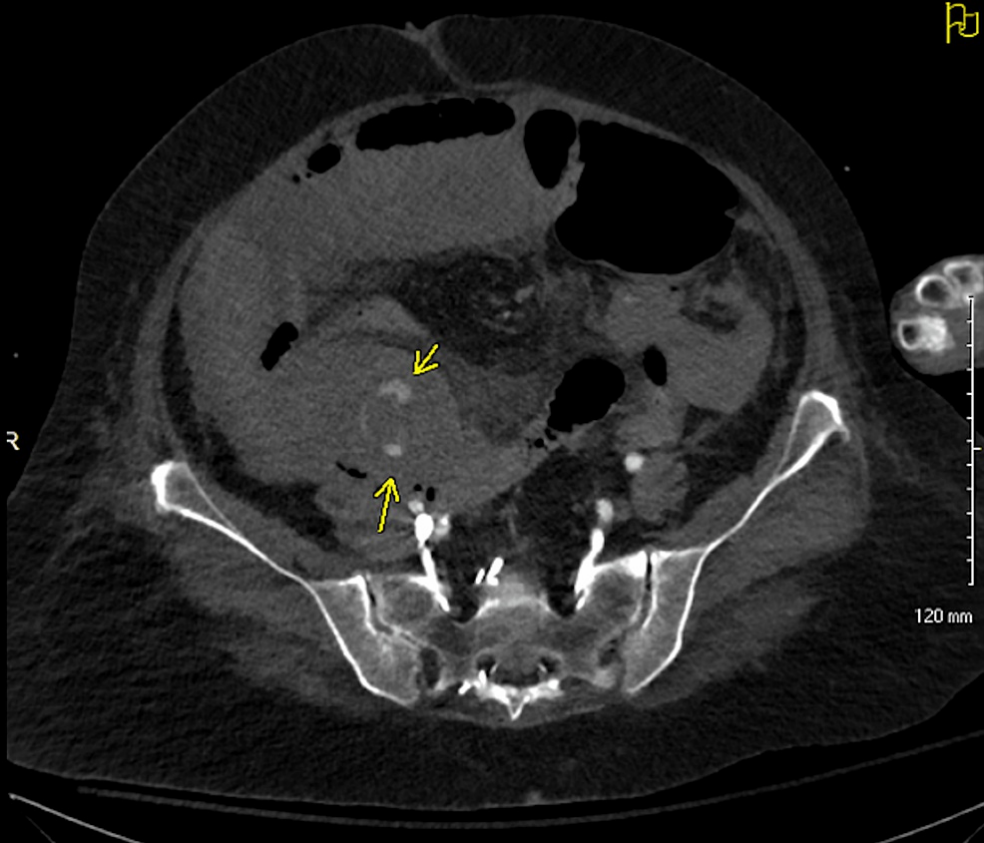
Contrast-enhanced (IV only) CT of the abdomen and pelvis (axial view) demonstrating evidence of intramural hematoma (arrow)

**Figure 2:**
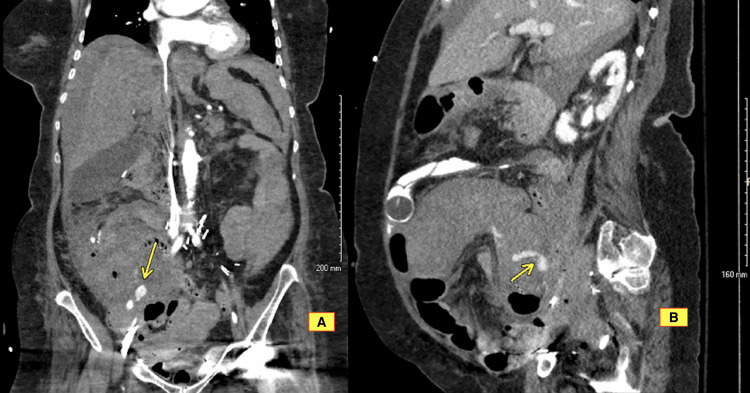
Contrast-enhanced (IV only) CT of the abdomen and pelvis showing postoperative changes with right hemicolectomy and right lower quadrant anastomosis with active contrast extravasation (arrow) at the anastomotic site compatible with active bleeding. (A) Coronal view. (B) Sagittal view.

A 78-year-old female presented to the emergency department with altered mentation, nausea, diarrhea, and dark-colored urine. Her medical history was notable for chronic opioid use, opioid-induced constipation, and recurrent urinary tract infection (UTI). She had bladder cancer for which she received radical cystectomy with neobladder formation using the right colon and appendix with concurrent right hemicolectomy and ileocolic anastomosis 17 years prior to this presentation. She had computed tomography (CT) of the abdomen and pelvis without contrast that showed non-specific wall thickening of the small bowel and colon. She was started on intravenous ceftriaxone and metronidazole for probable sepsis. The following day, she experienced hematochezia with clots. Laboratory results revealed a hemoglobin level of 10.9 g/dL (reference range: 14-18 g/dL), hematocrit of 31.7% (reference range: 42%-54%), platelet count of 106 K/µL (reference range: 150-450 K/µL), and an international normalized ratio (INR) of 1.7 (reference range: 0.9-1.1). She developed lethargy and hemodynamic instability with hypotension (70/45 mmHg) and tachycardia (115 bpm), prompting a need for emergency medical intervention including a massive transfusion protocol, vasopressor support, and mechanical ventilation. Initial abdominal CT angiography was inconclusive. She underwent emergent esophagogastroduodenoscopy (EGD) which did not reveal a source of GI bleeding. Colonoscopy showed blood in the rectum and recto-sigmoid junction, but the procedure was incomplete due to stenosis at the sigmoid colon. Repeat CT angiography demonstrated active contrast extravasation at the ileocolic anastomotic site compatible with active bleeding of the right external iliac artery (EIA) and hemoperitoneum (Figures 1, 2A, 2B).

**Figure 3:**
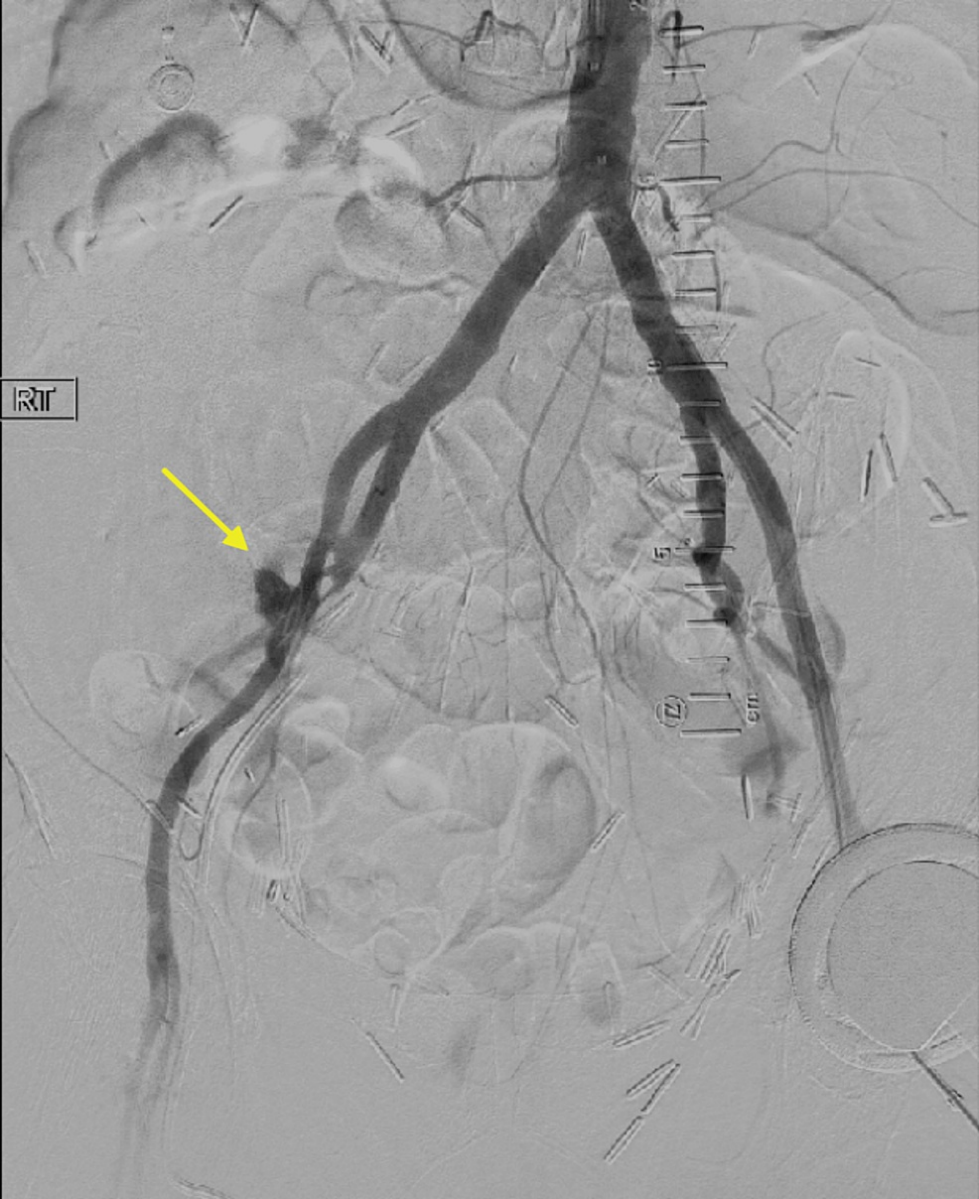
Active contrast extravasation (arrow) was observed from the mid right external iliac artery during bilateral iliofemoral arteriography prior to successful stent implantation

She underwent direct arteriography with stent placement of right EIA, which led to resolution of the bleeding (Figure 3).

Four days post-endovascular interventions, the patient developed multiorgan failure, sepsis secondary to Enterococcus faecalis (E. fecalis) bacteremia and disseminated intravascular coagulation and opted for comfort measures.

## Discussion

AEF is a rare condition, with about 0.007 incidences per million people [5]. AEF is classified into two types: primary and secondary. Primary AEFs occur in the absence of a history of previous aortic surgery or trauma, as opposed to secondary AEFs, which occur in the context of aortic repair surgery [6]. The main pathophysiology of fistula formation is constant irritation from aortic aneurysm pulsation and intestinal luminal stress leading to ulceration and focal necrosis, which progresses to complete fistulation [3]. Symptomatic presentation ranges from herald bleeding to hemorrhagic shock. Herald bleed is a small self-limiting hemorrhage that occurs hours prior to profound gastrointestinal bleeding and hemodynamic instability. The initial CT angiography did not reveal contrast extravasation. This is not unusual as during early stages, vasoconstriction and transient thrombus development may temporarily stabilize the patient causing negative or inconclusive findings on CT angiography. CT angiography has a sensitivity of 50% and a specificity of 100% [2].

Our case has a lot of distinctive features. Our patient had no prior aortic surgery; however, she did have a neobladder creation with right hemicolectomy and ileocolonic anastomosis 17 years before presentation. This demonstrates the significance of surgical history, as it might predispose a patient to catastrophic occurrences even years after surgery. Also, persistent opiate usage and constipation were important factors in our case that contributed to the development of AEF [7]. In our patient, chronic constipation resulted in elevated pressure over the anastomotic site from mechanical stress, which together with inflammation weakened the intestinal walls. This emphasizes the value of using opioids judiciously and addressing opioid-related constipation early. However, given the rarity and complexity of AEF, additional research is required in order to gain a deeper insight into the association between opioids, constipation, and the emergence of AEF.

Endovascular repair is becoming increasingly popular due to its minimally invasive approach to the treatment of AEFs [8]. Our patient underwent effective endovascular intervention, which included the implantation of a stent in the right IEA and the cessation of bleeding. It is important to keep in mind that endovascular repair is a stopgap measure before definitive surgical intervention. Patients remain at risk of developing septicemia because of the persisting fistula. Later in the hospital course, our patient developed sepsis as a result of E. faecalis bacteremia. She did not undergo surgical interventions and opted for comfort measures.

## Conclusions

Maintaining a high index of suspicion for AEF when caring for patients with significant gastrointestinal bleeding is critical, especially in patients with a history of abdominopelvic surgery. CT angiography of the abdomen and pelvis should be considered for diagnosis. For haemodynamically unstable patients, a team-based approach remains the gold standard, and endovascular management is beneficial in stopping the bleeding and as a bridge to definitive surgery.
